# Preventing Penile Retraction After Fournier’s Gangrene: Progressive-Tension Soft-Tissue Fixation for Penile Reconstruction

**DOI:** 10.7759/cureus.116609

**Published:** 2026-09-20

**Authors:** Tomas Maciulaitis, Patricija Glovackaite, Mykolas Udrys

**Affiliations:** 1 Faculty of Medicine, Institute of Clinical Medicine, Clinic of Rheumatology, Orthopedics, Traumatology and Reconstructive Surgery, Vilnius University, Vilnius, LTU; 2 Faculty of Medicine, Vilnius University, Vilnius, LTU

**Keywords:** case report, fournier's gangrene (fg), penile radix, penile reconstruction, penile retraction, skin graft fixation

## Abstract

Fournier’s gangrene (FG) is a rapidly progressive necrotizing soft-tissue infection associated with substantial morbidity and mortality. Extensive genital debridement may result in complex defects requiring reconstruction to restore function, body image, and quality of life. Penile reconstruction is particularly challenging because successful skin grafting requires both graft immobilization and maintenance of penile projection. We report a 58-year-old man with type II diabetes mellitus and obesity who presented with fever, genital pain, and severe systemic inflammation (C-reactive protein: 248 mg/L). Following intensive care admission, he underwent two surgical debridements. The scrotal soft tissues were completely excised, and the testes were preserved in bilateral subcutaneous anteromedial thigh pouches. After infection control, an extensive circumferential penile skin defect remained. Negative-pressure wound therapy (NPWT) was attempted; however, despite induction of an artificial erection, a reliable airtight seal could not be maintained because of abundant and highly mobile pubic tissues. To address the tendency toward proximal penile retraction, defined as displacement of the penile shaft into the surrounding mobile soft-tissue envelope, the pubic tissues were sequentially anchored circumferentially to Buck’s fascia using buried 4-0 polydioxanone (PDS) sutures in a progressive-tension fashion. Fixation points were tailored intraoperatively to progressively obliterate the dead space while preserving tissue perfusion. The shaft was subsequently resurfaced with an unmeshed split-thickness skin graft harvested from the right thigh. At one month, stable graft coverage and penile projection were maintained without recurrent retraction, and the patient was able to void spontaneously without difficulty. This case highlights that circumferential penile reconstruction may require stabilization not only of the graft-to-bed interface but also of the surrounding mobile soft-tissue envelope. Progressive-tension fixation to Buck’s fascia may provide a useful adjunct in selected patients in whom obesity, tissue mobility, and penile retraction complicate conventional graft stabilization.

## Introduction

Fournier’s gangrene (FG) is a rapidly progressive necrotizing soft-tissue infection of the genital, perineal, and perianal regions associated with substantial morbidity and mortality [1]. Prompt recognition, broad-spectrum antimicrobial therapy, hemodynamic support, and early, often repeated, surgical debridement are essential for survival [2]. Diabetes mellitus, obesity, advanced age, and immunosuppression are among the commonly reported predisposing factors [3,4]. Although aggressive debridement is fundamental to infection control, it may result in extensive genital soft-tissue defects requiring subsequent reconstruction [5].

Once the acute infection has been controlled, reconstructive management becomes important not only for wound closure but also for restoration of function, body image, psychological well-being, and quality of life [6]. The choice of reconstruction depends on the location, depth, and extent of tissue loss and may include secondary healing, primary closure, skin grafting, local or regional flaps, and testicular transposition into subcutaneous thigh pouches when scrotal coverage is unavailable [7]. Penile skin loss presents a particular reconstructive challenge because the replacement tissue must remain thin and pliable while accommodating changes in penile dimensions and preserving shaft mobility and projection [8].

Split-thickness skin grafting is a well-established option for extensive penile skin defects; however, successful reconstruction depends on more than graft selection alone [9]. The cylindrical and mobile nature of the penis makes graft immobilization difficult, and shear, fluid accumulation, and inadequate graft-to-bed contact may compromise graft take. Various methods, including tie-over dressings, external splinting, syringe-based fixation, and negative-pressure wound therapy (NPWT), have therefore been described to stabilize penile grafts [7]. Nevertheless, these techniques primarily address the graft-to-wound interface and may be technically difficult in patients with obesity and abundant mobile suprapubic tissues. In such patients, proximal retraction of the penile shaft into the surrounding soft-tissue envelope represents an additional reconstructive problem and should be distinguished from a pre-existing buried or concealed penis. [10]. Progressive-tension sutures, which sequentially anchor and advance mobile soft tissues to a fixed underlying tissue plane while progressively eliminating dead space, may offer a means of addressing this mechanical problem directly. We present a case of extensive penoscrotal soft-tissue loss following FG in an obese 58-year-old man, in whom negative-pressure graft stabilization was unsuccessful despite artificial erection because a reliable airtight seal could not be maintained. Penile reconstruction was achieved using circumferential progressive-tension fixation of the mobile pubic soft tissues to the penile shaft, sequentially obliterating the surrounding dead space and preventing proximal retraction, followed by resurfacing with an unmeshed split-thickness skin graft. This case highlights the importance of controlling the deeper soft-tissue envelope, in addition to graft immobilization, during reconstruction of complex circumferential penile defects.

## Case presentation

A 58-year-old man with a medical history of type II diabetes mellitus and obesity presented to the emergency department with fever and severe pain in the genital region. Laboratory investigations demonstrated a marked systemic inflammatory response, with a C-reactive protein level of 248 mg/L (normal range: 0-5 mg/L). FG was diagnosed, and because of the severity of his condition, the patient was admitted to the intensive care unit for resuscitation, broad-spectrum antimicrobial therapy, and surgical management. The patient underwent two consecutive surgical debridements performed by the urology team to achieve adequate source control and remove all nonviable tissue. Extensive necrosis necessitated complete excision of the scrotal soft-tissue envelope, leaving the testes without local soft-tissue coverage. Both testes were preserved and transposed into subcutaneous anteromedial thigh pouches. Following repeated debridement, control of the infection, and stabilization of the patient's general condition, he was referred to the Department of Plastic and Reconstructive Surgery for definitive reconstruction. At this stage, an extensive circumferential penile skin defect remained, with a viable wound bed suitable for skin grafting (Figure 1).

**Figure 1:**
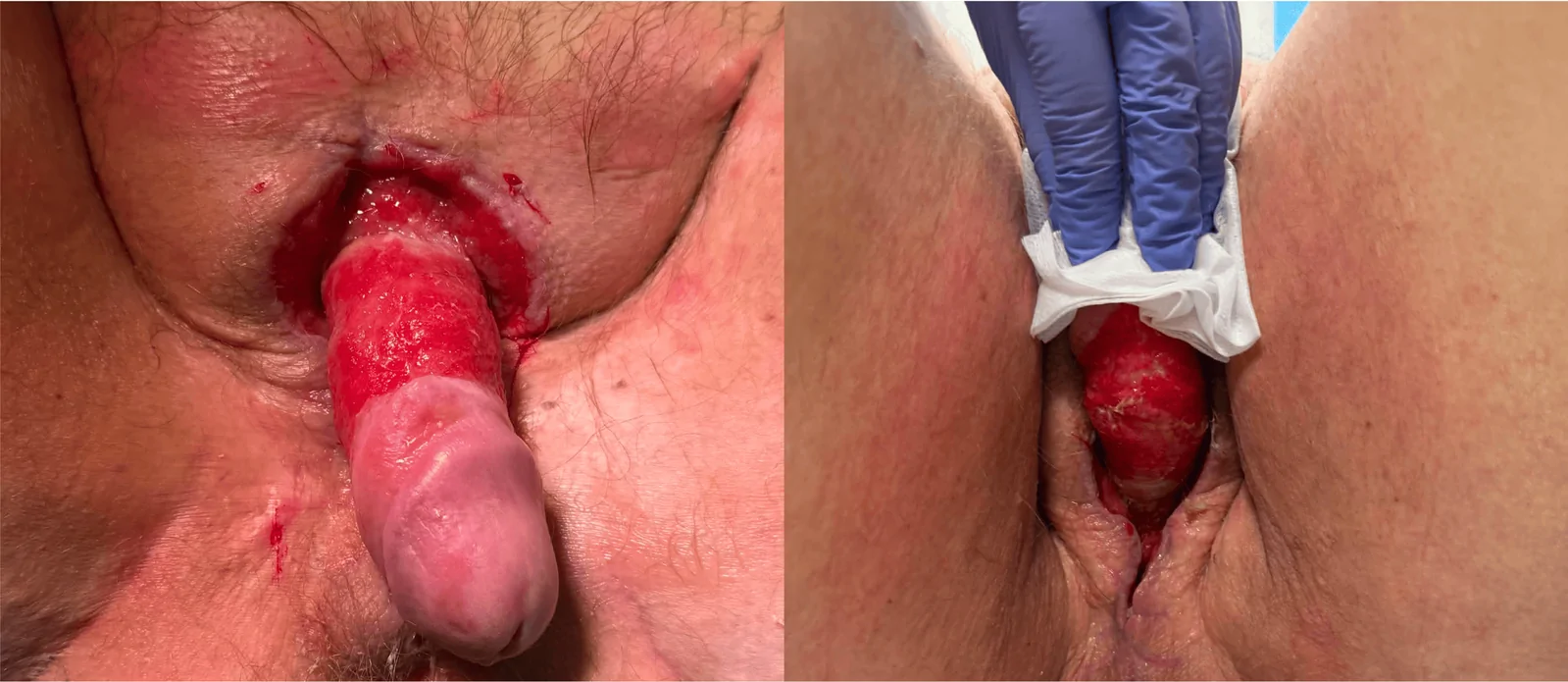
Penoscrotal defect following surgical debridement for Fournier’s gangrene

The patient's obesity and prominent suprapubic adiposity presented an additional reconstructive challenge. The abundant surrounding pubic soft tissues were highly mobile and tended to migrate around the penile base, allowing proximal retraction of the shaft. NPWT was initially attempted as a means of wound and subsequent graft stabilization. An artificial erection was induced to maximize penile projection and facilitate circumferential application of the negative-pressure dressing. Nevertheless, redundant, mobile surrounding tissues repeatedly folded around the penile base, preventing maintenance of a reliable airtight seal and effective negative pressure. NPWT was therefore abandoned.

Definitive reconstruction was designed to address the underlying tendency toward penile retraction before resurfacing the shaft. With the penis maintained in an extended position, the mobile pubic soft tissues were progressively advanced and anchored circumferentially to Buck's fascia using buried 4-0 polydioxanone (PDS) sutures. Fixation was performed sequentially and at multiple levels in a progressive-tension fashion, progressively reducing and ultimately obliterating the surrounding dead space (Figure 2). Rather than using predetermined circumferential fixation points, suture placement was tailored intraoperatively according to the distribution of residual dead space and the mobility of surrounding tissues. Each fixation point was selected to achieve adequate tissue advancement and stabilization while avoiding excessive tension or visible compromise of tissue perfusion. Particular caution was exercised during suture placement along the dorsal and ventral aspects of the shaft because the dorsal neurovascular structures and urethra lie deep to Buck's fascia. Sutures were placed superficially within the fascia without penetrating the deeper planes, thereby protecting these structures. Sequential fixation continued until the surrounding pubic soft-tissue envelope was securely approximated to the shaft and no residual potential space remained for proximal penile retraction. This created a stable penile base and maintained shaft projection without reliance on an external fixation device.

**Figure 2:**
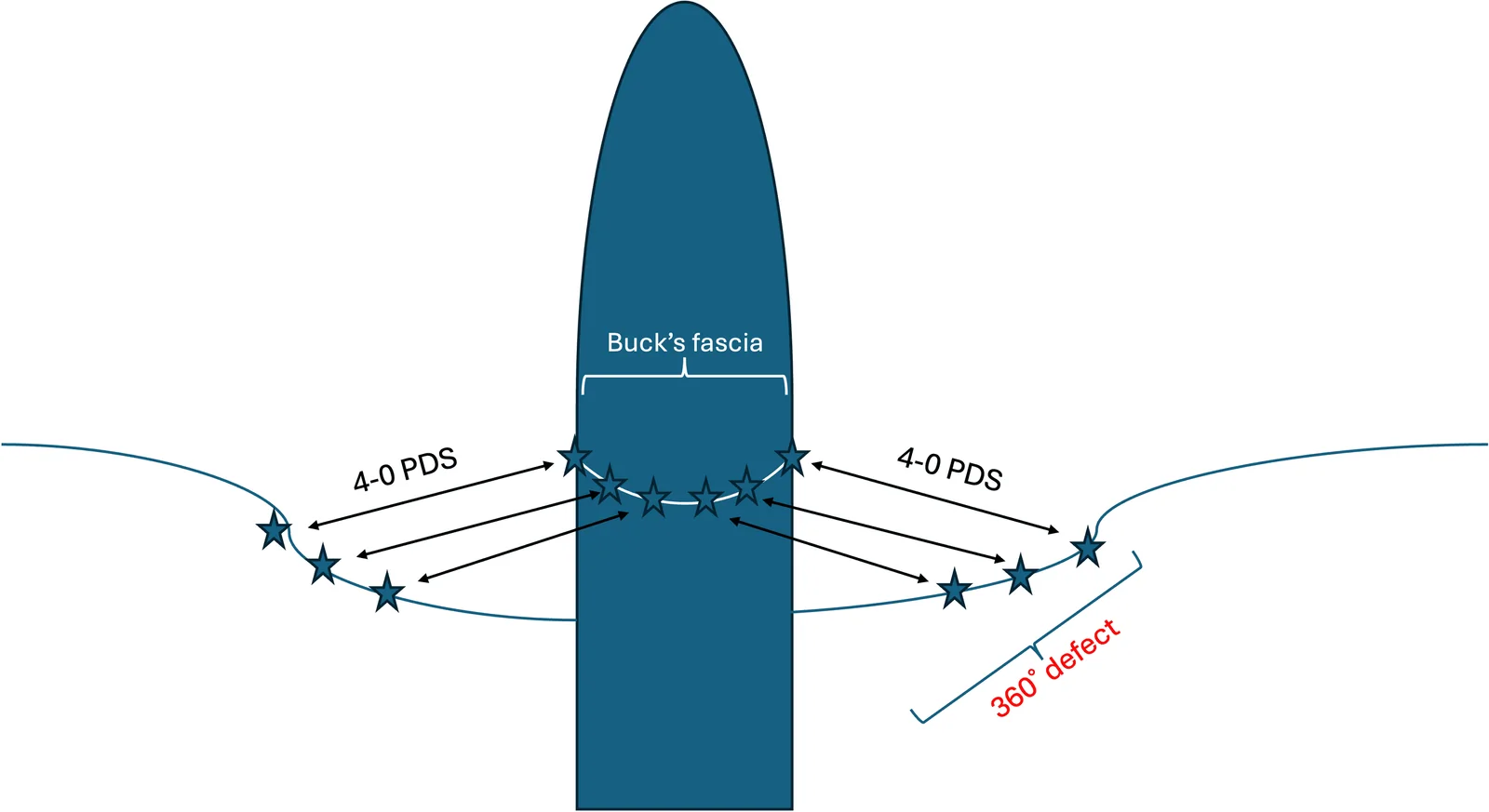
Schematic representation of the proposed progressive-tension fixation technique The schematic illustration was created by the authors using MS PowerPoint (Microsoft Corporation, Redmond, Washington, United States)

Once stable penile projection and complete dead-space obliteration had been achieved, the circumferential penile defect was resurfaced using an unmeshed split-thickness skin graft harvested from the right thigh with a dermatome. The graft was wrapped circumferentially around the shaft, with its junction positioned along the ventral midline to reproduce the anatomical appearance of the median raphe (Figure 3). It was secured to provide uniform graft-to-bed contact and covered with a nonadherent paraffin dressing. Following abandonment of NPWT, no rigid external splint or negative-pressure bolster was used; graft stability was achieved through direct fixation and the underlying stabilization of the pubic soft-tissue envelope.

**Figure 3:**
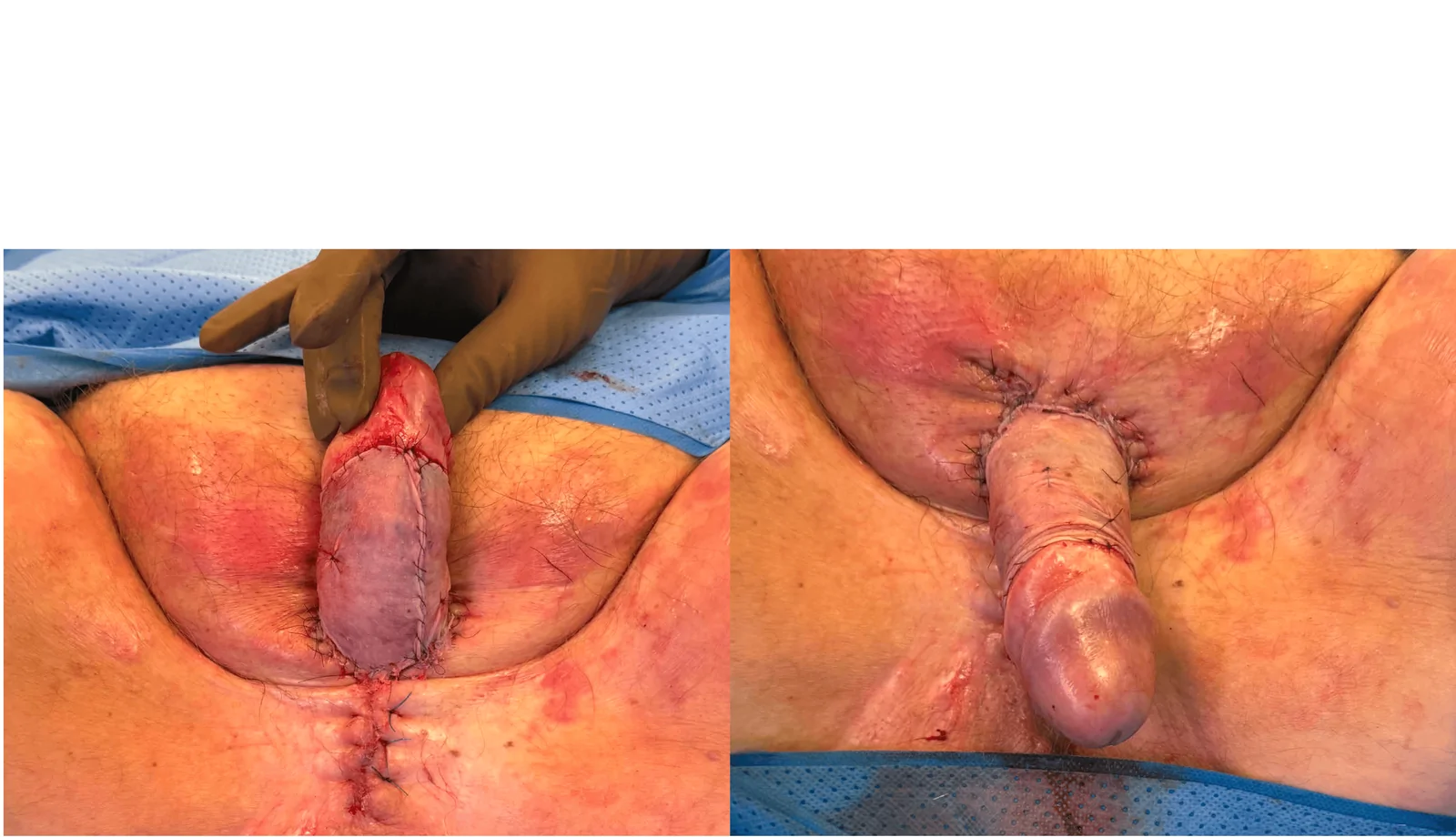
Completed circumferential penile reconstruction with progressive-tension soft-tissue fixation and split-thickness skin grafting

Postoperatively, the reconstruction was monitored for graft viability and stability. On postoperative day 5, the split-thickness skin grafting (STSG) demonstrated satisfactory adherence and take, with stable circumferential coverage of the penile shaft and no evidence of early proximal retraction or clinically significant dead-space formation. At the one-month follow-up, the graft remained viable with stable penile coverage and maintained shaft projection, without recurrent proximal retraction (Figure 4). The patient was able to void spontaneously without urinary difficulty. No additional reconstructive procedure was required during this period.

**Figure 4:**
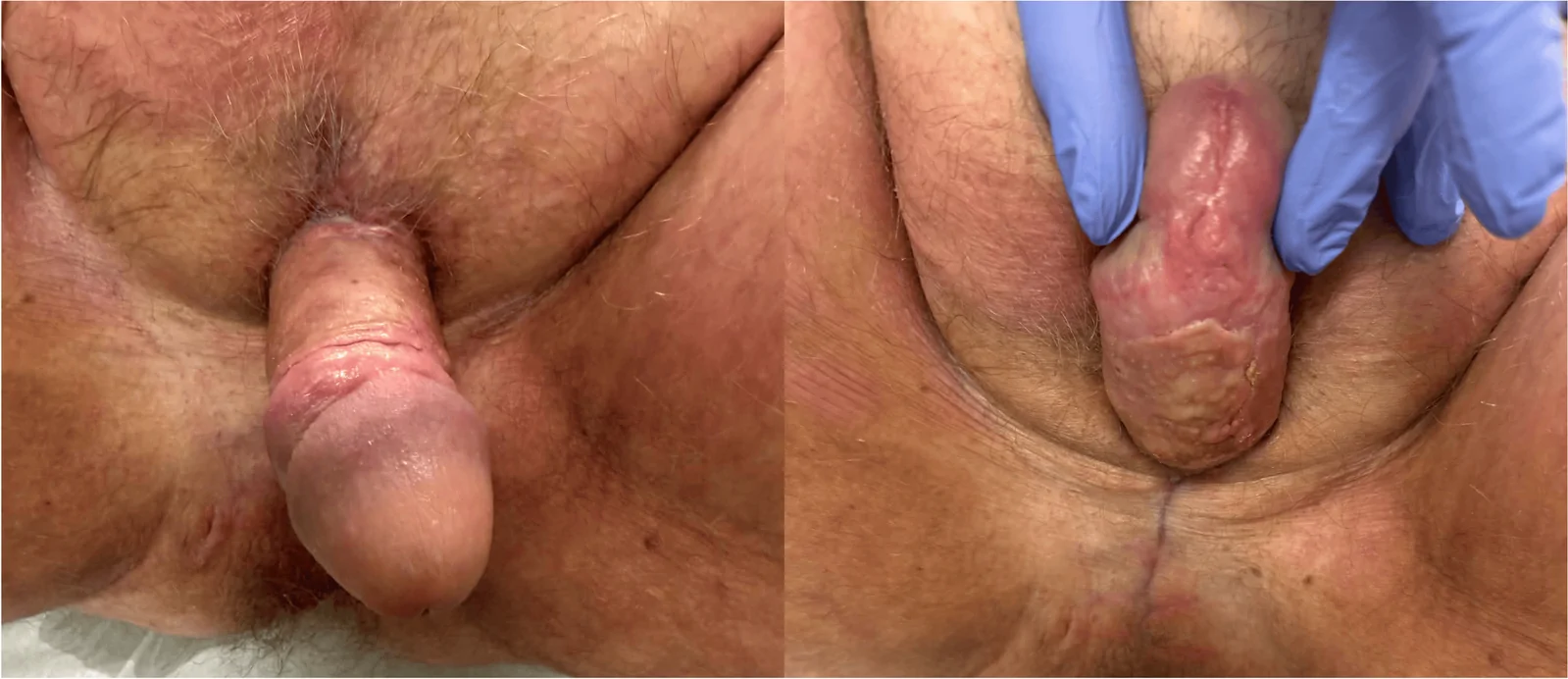
One-month postoperative outcome following penile reconstruction

## Discussion

Despite advances in multidisciplinary management, FG remains associated with substantial morbidity and mortality [11]. Reported outcomes vary according to patient comorbidities, disease severity, microbial etiology, and the timing of diagnosis and surgical intervention. In a large systematic review encompassing 3,646 patients, Shet et al. reported a pooled mortality rate of 20.4%, emphasizing the persistent lethality of the disease despite contemporary treatment [4]. Early recognition and prompt surgical source control are therefore essential, as delays in diagnosis and debridement have been associated with poorer outcomes [5]. Once the acute infection has been controlled, however, the clinical focus shifts toward management of the often extensive soft-tissue defects created by serial debridement. Long-term functional and aesthetic outcomes are influenced by the extent of tissue loss, number of procedures required, and availability of appropriate reconstructive strategies [12].

The importance of reconstruction should not be considered purely in terms of wound closure. Extensive genital tissue loss may profoundly alter body image, sexual identity, self-esteem, and quality of life. Recent literature has specifically identified reduced quality of life and self-esteem among survivors of FG, while systematic reviews of reconstructive techniques have highlighted the relative lack of patient-reported outcomes concerning cosmesis, function, and mobility [6,13]. These considerations are particularly relevant following complete loss of the scrotal soft-tissue envelope and extensive penile skin loss, as occurred in our patient. Reconstruction should therefore aim beyond uncomplicated wound healing and seek to restore an anatomically and functionally acceptable genital configuration whenever possible.

Management of the exposed testes after extensive scrotectomy represents one component of this reconstructive process. Subcutaneous thigh pouches have historically been used either as definitive coverage or as a temporary measure until subsequent reconstruction [14]. The technique is relatively simple and avoids additional complex reconstruction in a critically ill patient; however, concerns have been raised regarding appearance, discomfort, psychological effects, and the higher-temperature environment of the thigh, which may adversely affect testicular function [6]. In the present case, complete excision of the scrotal soft tissues necessitated bilateral transposition of the testes into subcutaneous anteromedial thigh pouches, protecting while the remaining genital wounds were managed and definitive penile reconstruction was planned.

For extensive penile skin loss with preservation of the underlying structures, STSG offers a relatively simple, single-stage method of providing thin and pliable coverage. Skin grafting is well-established in the reconstruction of large defects after FG and generally entails lower operative complexity and donor-site morbidity than flap reconstruction [15]. Flaps provide durable vascularized tissue and may reduce contracture, but they are thicker, involve greater operative complexity, and introduce additional donor- and recipient-site morbidity [6,7]. For penile resurfacing specifically, unexpanded STSGs have been advocated because they provide continuous coverage while avoiding the increased secondary contraction associated with expanded meshed grafts [12]. These considerations supported the use of an unmeshed STSG harvested from the thigh in our patient.

Successful penile grafting, however, depends not only on graft selection but also on reliable immobilization. Hematoma, infection, and shear are recognized causes of graft loss, and the flexible cylindrical recipient bed makes the penis a particularly challenging site for graft stabilization [16]. Multiple approaches have therefore been described, including bolsters, compressive dressings, penile splints, protective housings, traction devices, and NPWT [3,7,12]. NPWT is especially attractive because it provides close graft-to-bed contact, limits shear, and removes fluid accumulating beneath the graft. In a series of 13 patients undergoing graft reconstruction after FG, Orhan and Şenen reported no graft loss with negative-pressure fixation [17].

Nevertheless, penile reconstruction in patients with obesity presents an additional mechanical problem. Senchenkov et al. specifically identified obesity as a challenge to penile skin grafting because the penis may retract beneath or within the overlying abdominal fat pad [18]. They emphasized maintenance of penile stretch and immobilization as critical components of successful reconstruction and described a reinforced vacuum-assisted closure (VAC) construct designed to maintain the penis in an extended position during the first five postoperative days. This observation was particularly relevant in our patient, whose prominent suprapubic adiposity and abundant mobile pubic tissues created a tendency toward proximal penile retraction. Importantly, this problem involved not simply movement of the graft relative to its recipient bed but also movement of the penile shaft relative to the surrounding soft-tissue envelope.

NPWT was initially considered an appropriate solution in our patient because of its established ability to stabilize penile grafts. However, an effective airtight seal could not be maintained. Even after induction of an artificial erection to maximize penile projection, the abundant and highly mobile surrounding pubic tissues folded and shifted around the penile base, repeatedly compromising the seal. Although successful VAC-assisted penile grafting has been reported in obese patients, the technique may require additional rigid reinforcement to prevent collapse and wrinkling of the graft over a concealed or retractile penis [18]. Our experience illustrates that NPWT, although highly useful, may not be technically feasible in every patient and that the underlying tendency toward retraction must be addressed independently when the surrounding soft-tissue envelope is highly mobile.

Other external stabilization methods have also been described. Traditional approaches include rigid splints and bolsters, while syringe-based constructs have been proposed as a means of maintaining the penis under stretch during graft healing [6,19]. Such techniques primarily act externally to maintain graft contact and penile position. In our clinical experience, however, prolonged rigid fixation of the penis under tension may cause substantial patient discomfort and can become problematic during spontaneous erections. This observation should be interpreted cautiously because it is based on clinical experience rather than comparative evidence. Taken together, these limitations prompted consideration of whether the tendency toward retraction could instead be controlled directly at the level of the surrounding tissues.

The central technical principle of our reconstruction was therefore stabilization of the pubic soft-tissue envelope relative to the penile shaft before application of the skin graft. Rather than relying on an external device to maintain penile projection, the mobile pubic tissues were progressively advanced and anchored circumferentially to the penile shaft with multiple layers of buried 4-0 PDS sutures (Figure 2). Sequential fixation in a progressive-tension fashion gradually reduced and ultimately obliterated the surrounding dead space. This converted a mobile soft-tissue environment, in which the shaft could retract proximally, into a stable tissue envelope that maintained penile projection. The STSG was applied only after this deeper mechanical problem had been corrected.

An important technical consideration is the depth and distribution of fixation. In the present case, the progressive-tension sutures were anchored to Buck's fascia rather than placed indiscriminately into the penile shaft. Fixation points were selected intraoperatively according to the distribution of residual dead space and surrounding tissue mobility rather than according to a predetermined pattern. Sequential circumferential fixation was continued until the potential space had been eliminated, while the degree of tension was adjusted to avoid excessive compression and preserve tissue perfusion. Particular caution was exercised during dorsal and ventral suture placement, with superficial fascial bites used to avoid penetration into deeper planes containing the dorsal neurovascular structures and urethra. Thus, the objective was not maximal tissue tension, but the minimum fixation required to eliminate dead space and stabilize the surrounding soft-tissue envelope without compromising penile vascularity.

This distinction may be clinically relevant because conventional discussions of penile graft fixation predominantly focus on the graft-to-wound interface. Secure apposition at this interface is essential for revascularization and graft take, and failure results from shear, fluid accumulation, or infection. Our case suggests that, particularly in obese patients, a second interface should also be considered: the relationship between the penile shaft and the surrounding pubic soft tissues. Even an adequately secured graft may not prevent loss of penile projection if the shaft itself remains capable of retracting into a large mobile suprapubic soft-tissue envelope. Progressive-tension fixation addresses this deeper mechanical component by eliminating the potential space available for retraction rather than attempting to oppose retraction solely through an external dressing or splint.

The use of progressive-tension sutures in this setting should not be interpreted as a replacement for established methods of penile graft stabilization. NPWT remains an effective technique when a reliable seal can be obtained, and the available literature demonstrates excellent graft take with appropriately applied negative-pressure dressings [18]. Similarly, other bolsters and splinting techniques may be appropriate depending on anatomy and surgeon preference. Instead, the present technique represents an additional reconstructive strategy for patients in whom abundant mobile pubic tissues and a tendency toward penile concealment or retraction complicate conventional graft fixation. By addressing the surrounding tissue mechanics directly, it may also simplify subsequent graft stabilization.

At one month, the reconstruction remained stable, with maintained penile projection, viable graft coverage, and no clinically evident recurrent retraction. The patient was also able to void spontaneously without urinary difficulty. These findings extend the observation beyond initial graft take and provide preliminary evidence of short-term reconstructive stability. Nonetheless, this report remains limited by its single-patient design and relatively short follow-up. Longer observation is required to assess secondary graft contraction, durability of penile projection, and the potential need for revision. Erectile function, penile sensation, and patient-reported satisfaction were not formally evaluated during the available follow-up period. Accordingly, the present findings should be interpreted as demonstrating technical feasibility and a favorable short-term outcome rather than establishing superiority over established fixation methods.

## Conclusions

Reconstruction following extensive FG should aim beyond wound closure to restore stable genital anatomy and preserve function and quality of life. In this obese patient with abundant mobile suprapubic tissues, circumferential progressive-tension fixation to Buck's fascia enabled stepwise obliteration of the dead space and stabilization of penile projection before resurfacing with an unmeshed STSG. Stable graft coverage and penile projection were maintained at one month, with spontaneous voiding preserved. This case suggests that addressing the deeper soft-tissue mechanics may be a useful adjunct when conventional graft-stabilization methods are technically difficult. Further experience and longer follow-up are required to determine the durability and broader applicability of this approach.
